# Molecular and metabolomic effects of voluntary running wheel activity on skeletal muscle in late middle-aged rats

**DOI:** 10.14814/phy2.12319

**Published:** 2015-02-25

**Authors:** Sean M Garvey, David W Russ, Mary B Skelding, Janis E Dugle, Neile K Edens

**Affiliations:** 1 Abbott Nutrition R&DColumbus, Ohio, USA; 2 Division of Physical Therapy, Ohio UniversityAthens, Ohio, USA; 3 Ohio Musculoskeletal & Neurological Institute (OMNI), Heritage College of Osteopathic MedicineAthens, Ohio, USA

**Keywords:** Aging, exercise, metabolomics, muscle, rat, running wheel

## Abstract

We examined the molecular and metabolomic effects of voluntary running wheel activity in late middle-aged male Sprague Dawley rats (16–17 months). Rats were assigned either continuous voluntary running wheel access for 8 weeks (RW+) or cage-matched without running wheel access (RW−). The 9 RW+ rats averaged 83 m/day (range: 8–163 m), yet exhibited both 84% reduced individual body weight gain (4.3 g vs. 26.3 g, *P *=* *0.02) and 6.5% reduced individual average daily food intake (20.6 g vs. 22.0 g, *P *=* *0.09) over the 8 weeks. Hindlimb muscles were harvested following an overnight fast. Muscle weights and myofiber cross-sectional area showed no difference between groups. Western blots of gastrocnemius muscle lysates with a panel of antibodies suggest that running wheel activity improved oxidative metabolism (53% increase in PGC1*α*, *P *=* *0.03), increased autophagy (36% increase in LC3B-II/-I ratio, *P *=* *0.03), and modulated growth signaling (26% increase in myostatin, *P *=* *0.04). RW+ muscle also showed 43% increased glycogen phosphorylase expression (*P *=* *0.04) and 45% increased glycogen content (*P *=* *0.04). Metabolomic profiling of plantaris and soleus muscles indicated that even low-volume voluntary running wheel activity is associated with decreases in many long-chain fatty acids (e.g., palmitoleate, myristoleate, and eicosatrienoate) relative to RW− rats. Relative increases in acylcarnitines and acyl glycerophospholipids were also observed in RW+ plantaris. These data establish that even modest amounts of physical activity during late middle-age promote extensive metabolic remodeling of skeletal muscle.

## Introduction

Because of increasing sedentarism and overall age of western populations, it is of critical importance to identify lifestyle interventions from early to late middle age to lessen the negative consequences of inactivity and aging. Both caloric restriction and physical activity have been shown to promote longevity and healthspan, although significant controversy persists over the longevity-associated effects of caloric restriction in primates (Colman et al. 2009; Mattison et al. 2012). Nevertheless, caloric restriction and exercise “mimetics” are among the goals of several drug and natural product discovery programs targeting obesity, diabetes, cardiovascular disease, and aging (Baur et al. 2012; Mercken et al. 2012). However, in aging populations, the effect of these two interventions on body mass index (BMI) must be given extra scrutiny. Observational studies have shown that lower BMI (< 23 kg/m^2^) is associated with higher risk of mortality in elderly subjects (Winter et al. 2014), and that overweight subjects (BMI of 25 – <30), but not obese (BMI of ≥30), show lower risk of mortality than normal subjects (BMI of 18.5 – <25) (Flegal et al. 2013). These findings may be partly explained by the specific contribution of total lean body mass, or muscle mass, to healthy aging and lower risk of falls, disability, and disease. A threshold of muscle mass, and concomitantly a threshold of body weight, may be needed for optimal muscle function and overall health in elderly subjects.

Weight loss through physical activity is expected to more specifically drive fat loss while maintaining more lean body mass through biomechanical loading, than dieting alone. Thus, physical activity may provide a more optimal intervention for aging populations than simple caloric restriction. In middle-aged overweight and obese subjects, physical activity effectively promotes weight loss through both increased satiety response and reduced energy intake (King et al. 2009; Martins et al. 2010; Bales et al. 2012). Increased levels of physical activity and aerobic fitness are associated with lower risk of cardiovascular morbidity and mortality (Kodama et al. 2009; Samitz et al. 2011; Li and Siegrist 2012; Yates et al. 2013), decreased incidence of type 2 diabetes in high-risk individuals (Laaksonen et al. 2005), and even increased brain volume and improved cognitive function in elderly subjects (Larson et al. 2006; Erickson et al. 2010, 2011; Kulmala et al. 2014). Moreover, as little as 2.5 h of “non-vigorous” activity per week is associated with 19% reduced risk of mortality (Janssen 2011). Thus, modest low-volume physical activity, in combination with caloric maintenance, may provide an optimal stimulus for preserving, or improving, overall health without the reductions in nutrient intake or body weight that occur with caloric restriction (Morley et al. 2010; Weinheimer et al. 2010). Another advantage is reasonable probability of long-term compliance.

Many of the benefits of increased physical activity are facilitated by skeletal muscle, the maintenance of which is critical for health and functional independence with increasing age (Janssen 2011; Landi et al. 2012). Between the ages of 40 and 50 years, > 8% of muscle mass may be lost, and this number increases to >15% in decades after 75 years (Grimby et al. 1982; Janssen et al. 1985). Sarcopenia is a term used to describe severe loss of muscle mass and function with age (Clark and Manini 2008). It is encouraging that a modest increase in physical activity is associated with greater lean body mass in two independent longitudinal studies of elderly subjects (Shephard et al. 2013; Bann et al. 2014). Beyond protection of muscle size and function, physical activity-induced remodeling of muscle tissue may further contribute to overall health through both improved muscle insulin sensitivity (Wang et al. 1985; Ferrara et al. 2006) and the secretion of cardioprotective and neuroprotective myokines (Pedersen and Febbraio 2012). Further work is needed to identify specific biomarkers of and thresholds of physical activity sufficient for healthy aging. In particular, step count is a reasonable marker of physical activity that can be reliably and cost-effectively measured by small devices in community dwelling elderly (Storti et al. 2008; Harris et al. 2013). For these reasons, it is especially intriguing that Shephard et al. have proposed that a daily step count of 7000–8000 steps may protect against sarcopenia (Shephard et al. 2013).

The purpose of this study was to characterize molecular changes and metabolomic patterns in skeletal muscle following low-volume physical activity in a rat model of aging. A voluntary running wheel (RW) was used to model low-volume physical activity. Although older rats will voluntarily traverse as little as 10 meters per day, there is evidence to suggest positive biological impact of such small amounts of activity in rats (Cui et al. 2009; Novak et al. 2012; Russ et al. 2012). Preclinical rodent studies have also shown activity-associated improvement of many biological pathways predicted to enhance muscle function (Seburn and Gardiner 1985; Sexton 1995; Broch-Lips et al. 2011; Leiter et al. 2012; Novak et al. 2012; Pasini et al. 2012). Herein, we report the effects of 8 weeks of voluntary RW activity in late middle-aged male Sprague Dawley (SD) rats (16–17 months old). One experimental group was given continuous RW access for 8 weeks, while the second group was not allowed RW access. Age-related loss of skeletal muscle mass has been characterized in several rat inbred lines, and in particular, the impact on the myosin heavy chain (MHC) type II myofiber-predominant gastrocnemius muscle (Drew et al. 2003). With supplemental RW activity beyond cage activity alone, we predict that levels of markers of oxidative metabolism (PGC1*α*) and autophagy (LC3B-II/-I ratio) will increase in gastrocnemius muscle if, and only if, the level of physical activity afforded by RW access is sufficient. We also predict that intramuscular markers of hypertrophy (P70S6K, MSTN) and regeneration (PCNA, MYF5) will remain unchanged with the anticipated low volume of aerobic RW activity. We also sought to better understand aging muscle's metabolic response to RW activity by comprehensive metabolomic profiling of plantaris and soleus muscles. Altogether, these data help to define the therapeutic value of low-volume physical activity while highlighting novel metabolic markers of physical activity.

## Materials and Methods

### Experimental animals

All experimental procedures were approved by the Institutional Animal Care & Use Committees at both The Ohio State University (Columbus, OH) and Abbott Laboratories (Chicago, IL). Twenty male Sprague Dawley rats (16–17 months old) were purchased from Harlan (Indianapolis, IN). Rats were single-housed in Lafayette Instrument Company (Lafayette, IN) living chambers (model 80852, 48.3 × 26.7 × 20.3 cm) with Bed-o'Cobs® 100% natural corn cob bedding (The Anderson's Lab Bedding; Maumee, OH). All living chambers were attached to external activity wheels (Lafayette Instrument Company model 80850; 35.6 cm in diameter, hereafter referred to as running wheels, or RW) by way of a 5.7-cm-long tunnel. At receipt and throughout study, rats had ad libitum access to AIN-93M diet (Research Diets, #D10012M) and water. To facilitate acclimation to human handling, rats were handled and weighed on three separate days during the first 10 days. All rats were also allowed continuous access to running wheels for 3 weeks before the 3 days of baseline RW compliancy testing. Lights were on from 6:00 am to 6:00 pm.

### Grip strength

After 10 days of acclimation, baseline bilateral forelimb grip strength was determined using an automated grip strength meter (Order #1027DSR, Columbus Instruments, Columbus, OH). To facilitate compliant testing across multiple, consecutive trials, the experimenter's hand was placed under the belly of the rat and used to coax the rat's paws on to a triangular “pull bar” attached to a force transducer. The experimenter's alternate hand was used to pull back on the rat's tail. Peak tension (Newtons, N) calculated before the point of release was measured over five consecutive trials on any given day of testing. To allow for rat acclimation to the testing protocol, tension values from the first 2 days of baseline testing were discarded. Tension values from the following 4 days of testing were used to calculate mean baseline absolute grip strength (N) and baseline normalized grip strength (N/kg bw). Individual measurements were discarded if the rat did not have both forepaws engaged on the pull bar. A food lure was placed at the edge of the grip strength tester closest to the pull bar to further promote compliancy. In between consecutive tests, it was noted that placing the rat back in the cage for up to 5 sec resets volitional behavior leading to increased compliancy during subsequent trials. Baseline grip strength data were used to balance experimental groups; specifically, three poorly compliant rats were distributed such that they were not all assigned to the same experimental group. Grip strength was again measured at the end of study over five consecutive trials on any given day for 5 days. Only the first day served as the re-acclimation period, and values were recorded for the following 4 days. Baseline testing was not blinded to the tester. End of study testing was blinded to the tester. The same individual performed the grip strength testing at baseline and at the end of study. Three rats were discarded from end of study grip strength analysis due to forepaw and nail trauma, leaving seven rats per experimental group for all longitudinal analyses of forelimb grip strength.

### Running wheel activity

**Figure 1:**
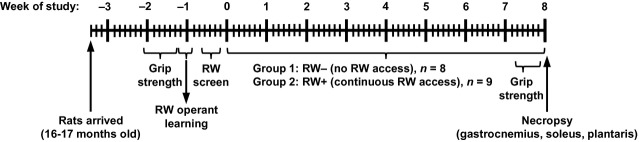
Overview of the voluntary running wheel (RW) study, including timeline of baseline and end of study testing. Directly following 3-day baseline RW activity screening, late middle-aged (16–17 months old) male Sprague Dawley rats were assigned to either no RW access (RW-) or continuous RW access (RW+) for 8 weeks.

Running wheel (RW) data, based on number of revolutions of the wheel, were collected using Lafayette Instrument Company's Activity Wheel Monitoring Software. Following baseline grip strength testing, rats were operantly conditioned to RW activity with treats once a day for 2 days. Following conditioning, a 3-day screen of baseline RW activity was conducted to facilitate balanced assignment of rats exhibiting low baseline RW activity to experimental groups. It was previously determined by our laboratory with a separate cohort of SD rats that 3 days of “pre-study” RW activity testing predicts chronic RW activity (S. M. Garvey, unpublished data). Three-day baseline data were only collected from 15 of the 20 rats, as technical difficulties left only 15 wheels transmitting data at the time. Both 3-day baseline RW activity and baseline grip strength data were used to evenly balance rats across the two experimental groups: Group 1) prohibited access to running wheels (RW−) and Group 2) continuous access to voluntary running wheels (RW+) (Fig.1). Note that experimental groups were not balanced for body weight. RW+ rats were then allowed continuous, unrestricted RW access for 56 days, while RW access was denied for the RW− rats with a sliding metal door placed in the closed position at the distal end of a small tunnel between the cage and the RW. Thus, the rats were thoroughly cage-matched, including the small tunnel. This is important because rats commonly repose in the small tunnel regardless of RW access. RW data were automatically recorded daily for 50 days. Three of the 20 rats died throughout the RW protocol. During end of study grip strength testing, RW+ rats continued to have access to wheels but distance data were not recorded for technical reasons. Data were downloaded manually on a weekly basis.

### Muscle morphometry

Following completion of the RW protocol, the remaining 17 rats were fasted for 16 h overnight prior to euthanasia by carbon dioxide asphyxiation, necropsy, and tissue collection. Heart, gastrocnemius, plantaris, and soleus muscles were removed and trimmed of all visible fat and connective tissue, blotted, and weighed. Muscles were wrapped in aluminum foil, frozen in liquid nitrogen, and stored at −80°C. For myofiber cross-sectional area (CSA) analysis, the lateral head of the left gastrocnemius was mounted onto cork board and then covered with Tissue-Tek® Optimal Cutting Temperature (O.C.T.) Compound (Sakura Finetek USA, Inc.; Torrance, CA). The specimen was then immersed in liquid nitrogen-cooled isopentane for up to 60 sec. O.C.T. compound-embedded samples were covered in foil and stored at −80°C. Left lateral gastrocnemius samples were then shipped on dry ice to Seventh Wave Laboratories (Chesterfield, MO) for blinded cryomicrotomy and myofiber CSA analysis. These muscles were transversally bisected at the midpoint and another cross section was cut 5-mm distal to the midpoint. The face corresponding to the midpoint was frozen to the cryomicrotome chuck, and the distal face was sectioned at approximately 8 *μ*m. Three sections were cut and mounted on a slide for each muscle. The sections were fixed in 95% ethanol for 5 min and subsequently immunoreacted with a laminin antibody (Novus Biologicals; Littleton, CO; 1:1000 dilution) followed by secondary antibody and 3,3′-diaminobenzidine (DAB) detection. The immunohistochemically stained slides were used to determine myofiber CSA and minimum Feret's diameter for each cross-sectioned gastrocnemius muscle sample. Using an Olympus BX-51 microscope and DP72 camera, seven image fields per muscle were acquired at 10× magnification. The images were stored as .tif files and loaded into calibrated Visiomorph™ software (Visiopharm; Hoersholm, Denmark) for segmentation. Image features were classified and processed to select the muscle fibers. Only complete cross-sectional profiles of muscle fibers were selected; incomplete profiles at the edge of the image were not selected. After the myofiber profiles in the images were segmented, the images were exported to ImageJ to determine the CSA and minimum Feret's diameter of each myofiber. The mean numbers of myofibers evaluated were 880 and 828 in the RW− and RW+ groups, respectively.

### Western blot

Frozen medial gastrocnemius (MG) samples were brought to 4°C, weighed, minced on cold glass, homogenized on ice-cold buffer (10 mm sodium phosphate, pH 7.2, 2 mm EDTA, 10 mm sodium azide, 120 mm sodium chloride, 1% NP-40, plus Halt Protease/Phosphatase Inhibitor Cocktail (Thermo Fisher Scientific Inc./Pierce; Rockford, IL)), incubated on ice for 1 h, and centrifuged at 14,000 g for 30 min, and supernatants were collected. Total protein concentrations were estimated using the Coomassie Plus (Bradford) Protein Assay (Thermo Fisher Scientific Inc./Pierce). Samples were diluted 1:1 with 2 ×  Laemmli sample buffer and 40 *μ*g protein were separated via SDS-PAGE on 10 or 12.5% gels with 5% stacking gels, at 150 V. Following SDS-PAGE, proteins were transferred to polyvinylidene fluoride (PVDF) for 2 h at 4°C in transfer buffer (15% methanol, 25 mm tris, and 192 mm glycine). Once transfer was complete, membranes were blocked at room temperature for 1 h in blocking buffer (Odyssey, LI-COR, Lincoln, NE), then incubated overnight at 4°C with primary antibodies. Antibodies against myogenic factor 5 (MYF5, #sc-302), proliferating cell nuclear antigen (PCNA, #sc-56), ribosomal protein S6 kinase, 70 kDa, polypeptide 1 (P70S6K, #sc-8148), phospho-P70S6K (Thr 421/Ser 424, #sc-7984), and myostatin (MSTN, #sc-6885) were purchased from Santa Cruz Biotechnology (Dallas, TX). Antibodies against protein kinase, AMP-activated, alpha 1 catalytic subunit (AMPK, #ab110036), phospho-AMPK (Thr172, #ab72845), phosphorylase, glycogen, muscle (PYGM, #ab88078), and vascular endothelial growth factor A (VEGFA, #ab1316) were purchased from Abcam (Cambridge, MA). The antibody against microtubule-associated protein 1 light chain 3 beta (LC3B, #TA301542) was purchased from OriGene (Rockville, MD). The antibody against peroxisome proliferator-activated receptor gamma, coactivator 1 alpha (PPARGC1A or PGC1*α*, #ST1202) was purchased from EMD Millipore/Calbiochem (Billerica, MA). All primary antibodies were diluted 1:2000 in blocking buffer plus Tween 20. After primary incubation, membranes were washed 5 × 5 min in tris-buffered saline plus Tween 20 (TBS-T) and incubated for 1 h at room temperature with appropriate secondary antibodies (LI-COR, Lincoln, NE) which were diluted in blocking buffer (1:10,000–1:20,000). Following secondary incubation, membranes were once again washed 5 × 5 min in TBS-T, then rinsed for 5 min with TBS. Membranes were dried in the dark overnight prior to scanning and densitometric band analysis with a LI-COR Odyssey system. After scanning, the membranes were stained with Coomassie Brilliant Blue R250 and within blot band intensities were normalized to total protein per lane determined from the stained, scanned membrane. The blots were all performed in duplicate. Known amounts of rabbit and mouse IgG were run on each gel as a standard for normalization of bands from different blots.

### Myosin heavy chain analysis

Myofibrillar proteins were extracted from the frozen MG samples, and used for electrophoretic determination of the relative expression of myosin heavy chain (MHC) isoforms as described previously (Russ et al. 2012). Gels were stained with Coomassie Brilliant Blue, scanned, and quantified densitometrically on a LI-COR Odyssey system.

### Muscle glycogen

One portion of the frozen MG was processed for spectrophotometric determination (absorbance at 490 nm) of glycogen content according to the method of Lo et al. (Lo et al. 1970). Glycogen content was normalized to the wet weight of the muscle sample.

### Global metabolomics

In sum, nine RW+ samples and eight RW− samples were included in the metabolomic analysis of plantaris and soleus muscles. Metabolomic profiling was performed by Metabolon (Durham, NC) according to the published methods (Evans et al. 2009). Following receipt at Metabolon, samples were extracted and normalized by weight. Extracts underwent chromatographic separation, followed by full-scan mass spectroscopy, to record and quantify all detectable ions presented in the samples. Biochemicals with known chemical structure were identified by matching the ions’ chromatographic retention index and mass spectral fragmentation signatures with reference library entries created from authentic standard biochemicals under the identical analytical procedure as the experimental samples. Raw area counts for each biochemical in each sample were normalized to correct for variation resulting from instrument interday tuning differences. Raw area counts for a compound were divided by the median value, setting the medians equal for each day's run. Missing values were assumed to result from areas being below the limits of detection. Thus, missing values for a given biochemical were imputed with the observed minimum after the normalization step. Semiquantitative values were derived from integrated raw detector counts of the mass spectrometers. Importantly, while peak area comparisons between samples represent relative amounts of each ion detected, different compounds and ions have different ionization potentials. To preserve all of the variation, yet allow compounds of widely different raw peak areas to be compared directly on a similar graphical scale, the normalized intensities were scaled by their median values for each compound.

For the analysis of metabolomic data, we applied a conservative reporting requirement described by Garvey et al. (2014). In sum, biochemicals had to be detected in at least 70% of each experimental group for semiquantitative reporting of relative ratios (i.e., fold of change) and statistical analysis. Thus, if biochemicals were detected in at least 70% of each group within a tissue of interest (i.e., at least six of the eight soleus samples), any “missing” data were imputed with the lowest detectable value from either the plantaris or soleus sample. For fold of change analysis, the values were scaled to the median intensity for that biochemical across the experimental groups and reported as relative ratios. The data were log transformed for Welch's two sample T-test to compare data obtained from the RW+ and RW− samples. Comparisons between groups were taken as significant when *P *≤* *0.05. A trend for significance was set at 0.05 <  *P *<* *0.10. For the 45 biochemicals that did not meet the semiquantitative reporting threshold in either or both muscles, a separate min/max value statistical analysis was performed as described in Garvey et al. (2014). Only one of these biochemicals, dimethylarginine, passed the min/max test (i.e., all values from the RW− group were higher than all values from the RW+ group).

### Statistical analysis

Statistical significance was set at *P *<* *0.05 in a *T*-test. All *T*-tests were unpaired with the exception of longitudinal grip strength analysis (paired). Variation is reported as standard error.

## Results

### Running wheel activity and grip strength testing

**Figure 2:**
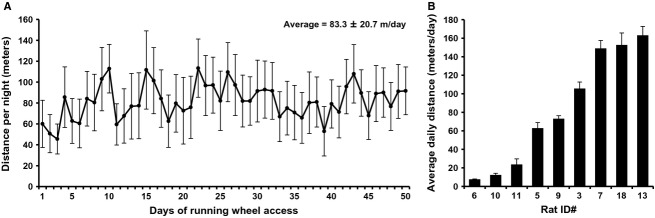
Daily running distances of rats with continuous access to voluntary running wheels (RW+) for 8 weeks. Values represent the mean ± SE of 9 rats (*A*) and mean ± SE of individual rats (*B*).

Voluntary RW distance was recorded daily for the first 50 days of the 8-week activity study period (i.e., RW protocol) (Fig.1). RW+ rats traversed an average of 83 m/day ± 21 m/day (Fig.2A). In contrast, young rats traverse an average of 3–18 km per day (Newhall et al. 1991). There was remarkable variability in RW activity between individual rats, with average daily distances ranging from 7.6 to 163.1 m/day (Fig.2B). Note that all rats that traversed < 25 m/day (i.e., “low runners”) during the 8-week RW protocol, which also underwent 3-day baseline RW screening, traversed < 25 m/day during baseline screening (data not shown). Thus, 3-day baseline RW screening is a useful method for evenly distributing low runners across experimental groups.

**Figure 3:**
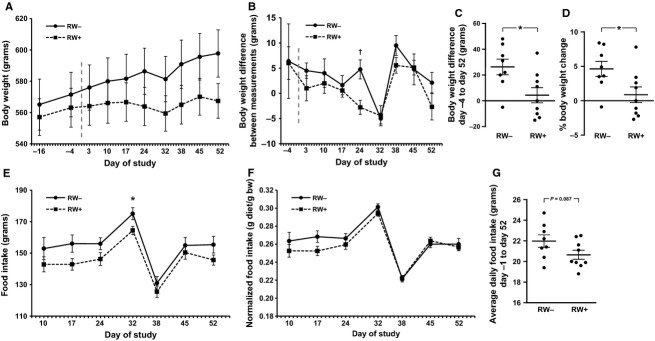
Body weight and food intake. Body weight was measured approximately weekly following receipt of animals and for 8 weeks following the start of the RW protocol (indicated by vertical hashed line) (A). Differences in consecutive body weight measurements (B). Absolute body weight differences (C) and percent body weight changes (D) between 4 days preceding and 52 days following onset of RW protocol. Approximately weekly food intake (E) and food intake normalized to body weight (F). Average daily food intake between 1 day preceding and 52 days following onset of RW protocol (*G*). *P <* *0.05, †*P *<* *0.01. Values represent the mean ± SE. Dot plots show values from individual rats; middle horizontal bars represent the mean ± SE (*C*,*D*,*G*). RW−, no running wheel access; RW+, continuous running wheel access.

Despite a trend for decreased absolute body weight with RW activity at the end of study, there was insufficient evidence for statistical significance (567 g ± 11 g vs. 598 g ± 15 g, *P *=* *0.12) (Fig.3A). There were, however, significant effects of RW activity on body weight gain. Compared to RW− rats, average individual rat body weight gain around weeks 3 and 4 was decreased in RW+ rats (−2.8 g vs. 4.8 g, *P *=* *0.005) (Fig.3B). Across the 8-week protocol, the RW+ group also exhibited reduced average (4.3 g ± 5.9 g vs. 26.3 g ± 6.0 g, *P *=* *0.020) and relative (0.9% ± 1.1% vs. 4.6% ± 1.1%; *P *=* *0.032) changes in body weight (Fig.3C and D).

RW activity was associated with a trend for lower food intake. Food intake was measured approximately every week on day −1 through day 52 of the RW protocol. Average food intake between any one measurement was not significantly different between RW+ and RW− groups, with the exception of the period between day 24 and day 32 when the RW+ group averaged 11 g less food consumption (164 ± 3 g vs. 175 ± 4 g, *P *=* *0.032) (Fig.3E). After normalization to body weight, there were no significant differences in food intake (Fig.3F). Across the 53 days of measurements, average daily food intake of the RW+ group was 1.4 g lower than the RW− group (20.6 ± 0.4 g vs. 22.0 ± 0.6 g, *P *=* *0.087) (Fig.3G).

**Figure 4:**
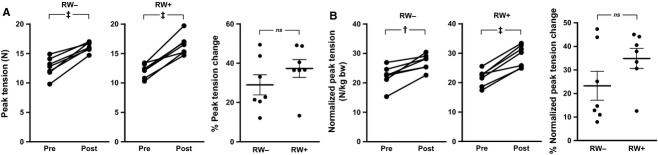
Bilateral forelimb grip strength testing. “Pre” and “post” study testing began on the 12th day before start of the RW protocol (d-12) and 52nd day of RW protocol (d52), respectively. Peak tension change (A, left), percent peak tension change (A, right), change in peak tension normalized to body weight (B, left), and percent normalized peak tension change (B, right). Values represent the daily mean across four consecutive days of testing (four tests per day). The horizontal bars in the dot plots represent the mean ± SE. †*P *<* *0.01, ‡*P *<* *0.001, *ns* not significant. RW−, no running wheel access; RW+, continuous running wheel access.

Bilateral forelimb grip strength improved in both experimental groups, yet no incremental enhancement was observed with RW activity (Fig.4). Compared to baseline peak tension (i.e., grip strength) measured on a horizontally fixed force transducer, an increase in peak tension after 8 weeks of study was observed in both RW− (12.7 ± 0.6 N vs. 16.1 ± 0.3 N, *P *<* *0.001) and RW+ (12.0 ± 0.4 N vs. 16.4 ± 0.6 N, *P *<* *0.001) groups (Fig.4A). The percent peak tension increases in RW+ and RW− groups were not significantly different (37.3% ± 4.5% vs. 29.0% ± 5.1%, *P *=* *0.25) (Fig.4A). Positive changes were also observed for peak tension normalized to body weight in both RW− (22.3 ± 1.4 N/kg vs. 27.0 ± 1.0 N/kg, *P *<* *0.01) and RW+ (21.6 ± 1.0 N/kg vs. 29.1 ± 1.4 N/kg, *P *<* *0.001) groups (Fig.4B). However, when comparing RW+ and RW− groups, the percent normalized peak tension increases were not significantly different (34.9% ± 4.2% vs. 23.3% ± 6.1%, *P *=* *0.14) (Fig.4B). Increasing muscle mass over the 2 months of study might explain these strength increases, or alternatively, extended acclimation to the animal facility and testing environment. Low baseline grip strength was the strongest predictor of change in grip strength (*R*^*2*^* *=* *0.60, *P *=* *0.001) across experimental groups (data not shown), highlighting the need to balance experimental groups for baseline grip strength in older rats.

### Muscle morphometry

Following overnight 16 h fasting, blood, skeletal muscle, and hearts were collected. RW activity had no effect on fasting blood glucose (Table1). There were no statistically significant differences in heart weight, normalized heart weight, absolute muscle wet weights, or normalized muscle wet weights between RW+ and RW− groups (Table1). There were also no differences in average gastrocnemius myofiber CSA or minimum Feret's diameter (Table1).

**Table 1:** Muscle morphometry

	RW− (*n *=* *8)	RW+ (*n *=* *9)
Fasting body weight (g)	572 ± 15	548 ± 12
Fasting glucose (mg/dL)	83 ± 2	84 ± 2
Wet weight (g)		
Gastrocnemius	3.22 ± 0.11	3.16 ± 0.25
Soleus	0.188 ± 0.008	0.176 ± 0.005
Plantaris	0.481 ± 0.026	0.507 ± 0.012
Heart	2.19 ± 0.12	2.11 ± 0.07
Normalized wet weight (mg/g bw)		
Gastrocnemius	5.64 ± 0.21	5.77 ± 0.10
Soleus	0.328 ± 0.013	0.321 ± 0.008
Plantaris	0.844 ± 0.050	0.928 ± 0.023
Heart	3.83 ± 0.60	3.86 ± 0.91
Gastrocnemius myofiber analysis		
Myofiber number counted	880 ± 55	828 ± 28
Cross-sectional area (*μ*m^2^)	3040 ± 182	3235 ± 99
Minimum Feret's diameter (*μ*m)	50.8 ± 1.6	52.4 ± 0.8

Values are means ± SE.

### Western blot

**Figure 5:**
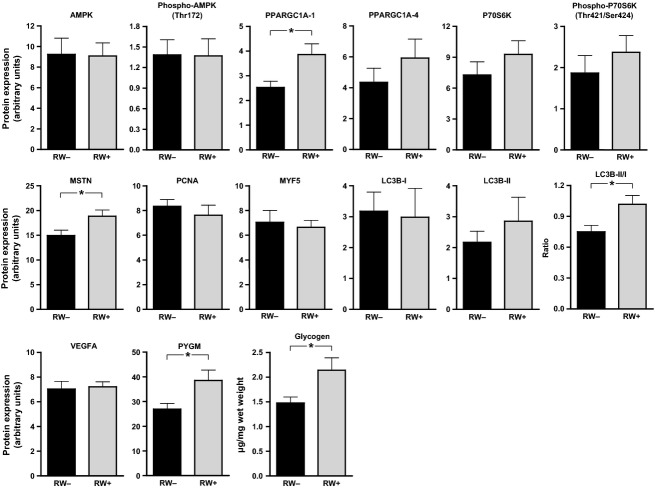
Western blot analysis and glycogen assay of medial gastrocnemius muscle. AMPK, AMP-activated protein kinase; PGC1A, peroxisome proliferator-activated receptor gamma coactivator 1 alpha; P70S6K, ribosomal protein S6 kinase, 70 kDa, polypeptide 1; LC3B, microtubule-associated protein 1 light chain 3 beta; MSTN, myostatin; PCNA, proliferating cell nuclear antigen; MYF5, myogenic factor 5; VEGFA, vascular endothelial growth factor A; PYGM, phosphorylase, glycogen, muscle.

The medial gastrocnemius muscles between RW− and RW+ groups exhibited several differences in protein expression, as determined by western blot analysis. No significant effects of RW access were found on the expression of protein kinase, AMP-activated, alpha 1 catalytic subunit (AMPK), phospho-AMPK or the ratio of phospho/total AMPK (Fig.5). Expression of the high molecular weight (∼110 kDa) isoform of peroxisome proliferator-activated receptor gamma, coactivator 1 alpha (PPARGC1A-1 or PGC1*α*-1) was increased 53% in the RW+ vs. the RW− group (*P *=* *0.027) (Fig.5). No changes were detected in the low molecular weight (∼38 kDa) isoform of PGC1*α* (PPARGC1A-4 or PGC1*α*-4). RW access did not affect the expression of ribosomal protein S6 kinase, 70 kDa, polypeptide 1 (P70S6K1), phospho-P70S6K1, the ratio of phospho/total P70S6K1, proliferating cell nuclear antigen (PCNA), or myogenic factor 5 (MYF5) (Fig5). However, the expression of myostatin (MSTN) was increased 26% in the RW+ group (*P *=* *0.036) (Fig5). RW access did not significantly alter expression of either microtubule-associated protein 1 light chain 3 beta, form I (LC3B-I, the inactive form) or form II (LC3B-II, the lipidated, active, autophagosomal form), although the nonsignificant changes that did occur went in opposite directions (i.e., RW access decreased LC3B-I and increased LC3B-II). As a result, the ratio of LC3B-II/-I was 36% higher in the RW+ group (*P *=* *0.027) (Fig.5). It has been suggested that this ratio is a better marker for autophagy than either the LC3B-I or -II form alone (Wohlgemuth et al. 2010). RW access did not alter the expression of vascular endothelial growth factor A (VEGF) (Fig5). Muscle glycogen phosphorylase (PYGM) expression was increased 43% in the RW+ vs. the RW− group (*P *=* *0.035) (Fig.5).

There were no significant differences in any of the myosin heavy chain (MHC) isoforms between the RW− and RW+ groups (data not shown). However, fasting muscle glycogen was 45% greater in the RW+ vs. the RW− group (*P *=* *0.038) (Fig.5).

### Global metabolic profile of skeletal muscles

We analyzed the global metabolic profile of the plantaris and soleus muscles. We detected 328 biochemicals in plantaris and 329 biochemicals in soleus. Of these biochemicals, 299 biochemicals in plantaris and 303 in soleus met the reporting threshold for semiquantitative analysis (i.e., the biochemical was detected in at least 70% of samples within each experimental group). Of these biochemicals, 253 biochemicals in plantaris and 251 in soleus matched known chemical structures in Metabolon's chemical reference library.

**Figure 6:**
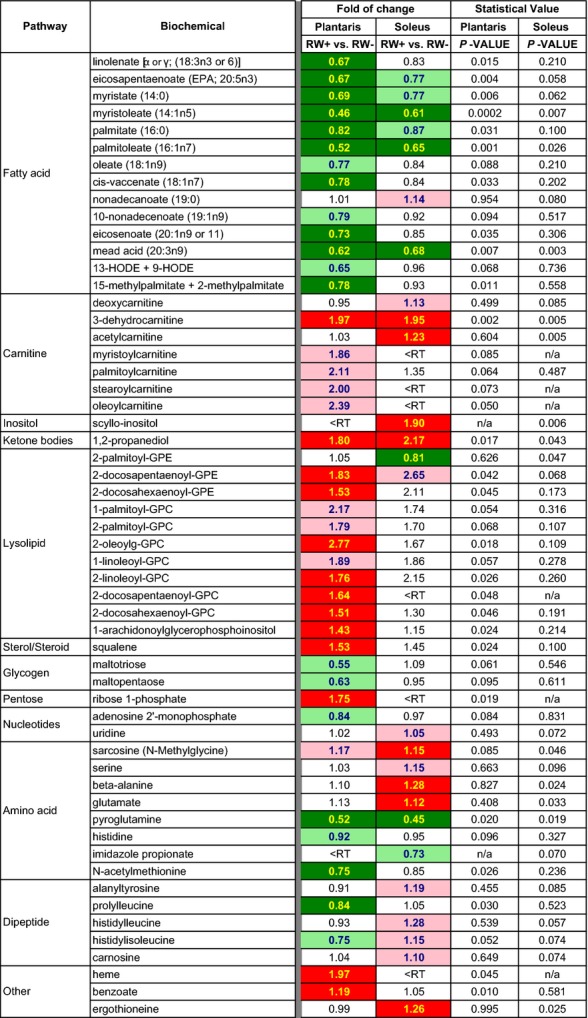
Summary of results from the metabolomic study. The heat map shows the relative ratios, or fold of change values, between biochemicals detected in the RW+ and RW− groups from plantaris and soleus muscles in 18- to 19-month-old SD rats. Green and red shading indicate statistically significant (*P *<* *0.05) decreases or increases in fold of change, respectively, in the RW+ group compared to the RW− group. Pink and light green shading indicate a trend (0.1 > *P *>* *0.5) for increases or decreases, respectively. <RT, values from at least one experimental group for given muscle do not meet 70% fill reporting threshold, i.e., at least six of eight rats in the RW− group or seven of nine rats in the RW+ group. *P*, *P*-value determined by Welch's *T*-test.

The biochemicals which met the 70% reporting threshold and their statistical comparisons between the RW+ and RW− muscles are presented in Table S1. Approximately 10% of the known biochemicals (*n *=* *26) in plantaris and 5% of the known biochemicals (*n *=* *13) in soleus showed significant RW+ vs. RW− differences by Welch's two-sample T-test (*P *≤* *0.05, Fig.6). An additional 16 and 13 biochemicals in plantaris and soleus, respectively, showed a trend (0.05 <  *P *<* *0.10) for effect of RW access. A separate min/max analysis was applied to the 45 biochemicals for which >30% of samples showed no detectable value within either experimental group (see Methods).

The majority of metabolomic changes with RW access were specific to lipid metabolism. Shown in Figure6 are the biochemicals that showed at least a trend for significance (*P *<* *0.10) in either plantaris or soleus muscles. In the plantaris muscle, 10 of the 25 detected long-chain fatty acid (LCFA) species (including essential and branched FAs) were significantly reduced between 18% and 54% in RW+ vs. RW− rats (Fig.6 and Table S1). Conversely, four of the six long-chain acylcarnitines detected in RW+ plantaris showed a trend for increased fold of change compared to RW− plantaris (i.e., myristoylcarnitine, 1.9-fold; palmitoylcarnitine, 2.1-fold; stearoylcarnitine, 2.0-fold; oleoylcarnitine, 2.39-fold). Free carnitine levels were not different between RW+ and RW− muscles (Table S1). Seven of the 27 detected lysolipids showed a significantly increased fold of change, ranging from 1.4- to 2.8-fold change, in RW+ vs. RW− plantaris. RW+ plantaris also showed an 80% increase in the ketone body, 1,2-propanediol (Fig.6); however, there was no change in 3-hydroxybutyrate (Table S1). Significant differences with RW access were also noted for pyroglutamine (−48%), N-acetylmethionine (−25%), prolylleucine (−16%), 3-dehyrocarnitine (+97%), squalene (+53%), ribose 1-phosphate (+75%), heme (+97%), and benzoate (+19%) in plantaris.

Far fewer metabolomics changes were observed in the soleus muscle between RW+ and RW− rats; however, these changes also largely reflected altered lipid metabolism (Fig.6 and Table S1). Three of the 25 LCFAs were significantly reduced in RW+ soleus (i.e., myristoleate, −39%; palmitoleate, −35%; and eicosatrienoate, −32%, also known as mead acid). Three additional LCFAs showed a trend for decreased fold of change, and only one of the 23 LCFAs (i.e., nonadecanoate, +14%) showed a trend for positive fold of change. Only three of the six acylcarnitines detected in plantaris met the reporting threshold in soleus, and there was no change with RW status. Acetylcarnitine, however, did show a 23% relative increase in RW+ soleus. Similar to plantaris, 1,2 propanediol was increased 2.2-fold in RW+ vs. RW− soleus, with no change in 3-hydroxybutyrate. Abundance of lysolipids was largely unaffected by RW activity in the soleus muscle, with the exception of 2-palmitoylglycerophosphoethanolamine (−19%). With regard to amino acid metabolism in soleus, RW activity promoted significant increases in sarcosine (+15%), beta-alanine (+28%), glutamate (+12%), and also showed a trend for increased fold of change of alanine or histidine-containing dipeptides (i.e., alanytyrosine, +19%; histidylleucine, +28%; histidylisoleucine, 15%; and carnosine, 10%). RW+ soleus also showed changes in 3-dehydrocarnitine (+95%), scyllo-inositol (+90%), pyroglutamine (−55%), and ergrothioneine (+26%).

## Discussion

This study tested the molecular and metabolomic effects of physical activity, assessed via voluntary running wheel (RW) activity, in late middle-aged SD rats. We found that 8 weeks of RW activity significantly decreased body weight gain compared to rats without RW access (Fig.3), although there were no significant effects on the weights of several hindlimb muscles (Table1). Although body composition was not directly measured, RW activity likely improved body composition through reduction of fat mass gain. As expected, RW activity increased levels of the high molecular weight form of PGC1*α*, the ratio of LC3B-II/-I, and glycogen phosphorylase (Fig.5). Consistent with enhanced beta-oxidation of lipids, RW activity was associated with a fasting intramuscular metabolomic signature comprising decreased abundance of long-chain fatty acids (LCFAs).

The import of these data does not derive so much from their novelty around exercise, but rather, to the remarkable effects so little physical activity produced. Young rats traverse an average of 3–18 km per day (Newhall et al. 1991), and RW activity progressively decreases with advancing age (Peng et al. 1980; Mondon et al. 1985; Russ et al. 2012). In this study, late middle-aged rats with RW access averaged only 83 m/day (Fig.2). The accompanying significant reduction in body weight gain may have been facilitated by mild caloric reduction, evidenced by a trend for reduced food intake (Fig.3). However, in a chronic RW study, sedentary pair-fed rats weighed approximately 25% more than rats with RW access (Holloszy et al. 1985), suggesting that RW access drives the lack of body weight gain. Pair feeding in future preclinical studies could help decode the acute interplay between caloric intake and physical activity on weight gain. Note that rats in this study likely exhibited acute changes in RW activity, food, and water intake that led to the significant change in weekly body weight at day 24 of the study (Fig.3B). These data are consistent with the irregular RW behavior of older rats, or alternatively, reflect an undocumented, acutely disruptive auditory or olfactory stimulus within the animal facility. Nonetheless, future studies may better standardize the low-volume activity stimulus across individual rats and across the duration of the study, whether by use of monitored treadmill running or voluntary running wheels that lock beyond a daily threshold of distance.

In young SD rats, RW activity is associated with an increase in soleus mass (Rodnick et al. 1985; Newhall et al. 1991), a shift away from type IIB myofibers in the plantaris (Kariya et al. 2004), and increased protein synthesis in both muscles (Munoz et al. 1994). Others have observed an increase in soleus weight in old rats (27- vs. 9-month-old female Long-Evans rats), however, with onset of RW activity early in life at 4 months of age (Brown et al. 1992). With onset of RW activity in late middle age, we did not observe any significant differences in hindlimb muscle weights or gastrocnemius myofiber CSA of SD male rats (Table1). In addition, both RW− and RW+ groups exhibited a comparable increase in forelimb grip strength (Fig.4), and analysis of MHC isoforms revealed no differences between RW− and RW+ groups (data not shown). Thus, the 8-week duration of RW activity applied at 16–17 months of age in this study may not have delivered a strong enough metabolic or biomechanical stimulus for MHC isoform modulation or myohypertrophy. A number of studies indicate that the hypertrophic response of muscle may be blunted with aging (Degens and Alway 2003; Martel et al. 2006). It may also be the case that the same duration of activity applied at an earlier age could promote persistent myohypertrophy throughout older age, as suggested by studies of testosterone injections at early middle age in mice (Egner et al. 2013). This hypothesis is also supported by a study of male SD rats, wherein plantaris MHC type IIx myofiber hypertrophy requires onset of RW activity before middle age (Kariya et al. 2004).

The effects of RW activity on several training adaptation-related myocellular processes were analyzed by western blot. Markers of two important signaling pathways, AMPK and P70S6K1, appeared unaffected by RW activity (Fig.5). However, many of the effects of these two proteins are related to acute activation, which is usually assessed via phosphorylation status of the proteins, a fairly short-lived modification for AMPK (Lee-Young et al. 2008). In this study, animals were not tested shortly after exercise, although AMPK phosphorylation would likely be acutely activated by a bout of RW activity. It has also been suggested that the effects of targets downstream of AMPK and P70S6K1 are the result of temporal summation of pathway activation in response to repetition of exercise over time (Winder et al. 2006). In this study, the RW activity-associated increases in PGC1*α* and the LC3B-II/-I ratio (Fig.5) are consistent with regular activation of AMPK, as the proteins are markers of mitochondrial biogenesis (Lin et al. 2002) and autophagy (Wohlgemuth et al. 2010), respectively, and both of these processes have been shown to be AMPK dependent (Pauly et al. 2012; Sanchez et al. 2012b; O'Neill et al. 2013). However, the possibility of AMPK-independent mechanisms remains open, particularly in the case of autophagy (Grotemeier et al. 2010). AMPK and P70S6K are thought to be parts of antagonistic pathways (Sanchez et al. 2012a), and the low-intensity, aerobic exercise in this study was unlikely to have stimulated the hypertrophy-related pathways associated with P70S6K1.

Low-volume RW activity was associated with an increase in myostatin protein (Fig.5). Myostatin is a secreted, skeletal muscle-specific cytokine that is responsible for negative regulation of muscle mass, hypertrophy, and regeneration. Several critical questions remain around myostatin inhibition, including whether a gain in muscle mass through myostatin reduction necessarily contributes to greater muscle strength. Myostatin-deficient mice grow skeletal muscles two to three times larger than littermate controls, yet with lessened muscle quality (Mendias et al. 2011). We propose that the increase in myostatin with RW activity in this study contributed to enhanced metabolism and mitochondrial biogenesis through a sparing of energy that would otherwise be used for muscle growth. In agreement with this hypothesis, RW+ rats showed no evidence of muscle weight gain (Table1). In conflict with our data, though, 6 months of moderate aerobic exercise training in middle-aged human subjects was associated with a decrease in both intramuscular and circulating myostatin (Hittel et al. 2010). The combination of overnight fasting and RW activity may have contributed to the increase in myostatin in our study.

PGC1*α* is a well-recognized mediator of mitochondrial activity and biogenesis in skeletal muscle, including in response to exercise (Lin et al. 2002; St-Pierre et al. 2003). PGC1*α* was also specifically upregulated in the plantaris muscle of young female mice following 4, 6, and 8 weeks of voluntary RW activity (8–12 km/day) (Ikeda et al. 2006). In this study, the older rats exhibited a comparable increase in PGC1*α* in the gastrocnemius muscle (∼50% in this study (Fig.5) vs. ∼30% in (Ikeda et al. 2006)), despite only running an average of 83 m/day (Fig.2A). Thus, we project that even modest levels of aerobic exercise in older adults can foster muscle health through PGC1*α*-mediated enrichment of mitochondrial number and quality. Note that the increase in PGC1*α* in the mouse study was not observed in soleus and tibialis muscles (Ikeda et al. 2006). Physical activity may better enhance mitochondrial adaptation in a subset of glycolytic, fast-twitch myofibers that make up the bulk of plantaris and gastrocnemius. There is mounting evidence that fast-twitch myofibers preferentially atrophy with age compared to slow-twitch myofibers (Joseph et al. 2012; Nilwik et al. 2013).

There may be a link between increased PGC1*α* levels and the RW activity-associated metabolomic profile comprising decreased long-chain fatty acids (LCFAs) and increased acylcarnitines (Fig.6). PGC1*α* has been shown to activate carnitine palmitoyltransferase (CPT-I) in liver (Louet et al. 2002; Song et al. 2010). CPT-I facilitates the rate controlling step in mitochondrial oxidation of LCFAs. CPT-I transfers the acyl moiety from fatty acyl-CoA to carnitine for the translocation of LCFAs across the mitochondrial membrane. There are at least three isoforms of CPT-I including a liver isoform CPT-Ialpha, a muscle isoform CPT-Ibeta, and a brain isoform CPT-Ic. Little is known about CPT in muscle, where its activity might enhance beta-oxidation of lipids, as suggested by the decrease in muscle LCFAs (Fig.6). Improved lipid beta-oxidation in RW+ rats could also be mediated by increased mitochondrial number or enhanced mitochondrial quality, both of which are implicated by increased PGC1*α* expression and increased ratio of LC3B-II/-I forms. Since all rats were fasted overnight, the interaction of energy depletion with physical activity must also be considered. Increased physical activity enhanced lipid beta-oxidation following overnight fast, an acute metabolic stressor which promotes lipid catabolism. It would be interesting to test whether the observed differences following overnight fast are also seen in the fed state, or whether a metabolic stressor (i.e., fasting) is needed to uncover the improved mitochondrial performance afforded by increased physical activity.

It is intriguing that 1,2-propanediol (propylene glycol) is increased in plantaris and soleus with wheel running (Fig.6). 1,2-propanediol is produced in vivo by the enzymatic conversion of acetone, which has largely been described in liver tissue (Casazza et al. 1984). Acetone occurs naturally as a ketone body produced when acetyl-CoA is in excess, commonly observed during carbohydrate depletion, prolonged fasting, and insulin resistance. The enzyme, cytochrome P450, family 2, subfamily e, polypeptide 1 (CYP2E1) which converts acetone to 1,2-propanediol, is predominantly expressed in liver with evidence for ethanol-induced kidney expression, fasting-induced white adipose tissue expression, and high-fat diet-induced liver expression (Porter et al. 1989; Yoo et al. 1991; Yoshinari et al. 2004); however, skeletal muscle expression has not been consistently observed (Crosbie et al. 1997; Smith et al. 2000). The presence of increased ketone bodies may indicate that more LCFAs are being mobilized than can be completely oxidized in response to RW activity. This hypothesis is in agreement with the increased levels of long-chain fatty acylcarnitines in plantaris muscle (Fig.6), awaiting either mitochondrial translocation or intramitochondrial oxidation. Increased acylcarnitines with RW activity was somewhat surprising, although, since these intermediates have been linked to insulin resistance and aging (Koves et al. 2008; Noland et al. 2009). In a fructose-fed rat model of type 2 diabetes mellitus, increased acylcarnitines in the glycolytic extensor digitorum longus (EDL) muscle were associated with decreased mitochondrial respiration in permeabilized fibers (Warren et al. 2014). The oxidative soleus muscle, on the other hand, exhibited decreased acylcarnitines, yet rates of respiration on par with rats fed a control diet (Warren et al. 2014). Thus, perhaps it is no surprise after all that the increase in acylcarnitines with RW activity was specific to plantaris, while acylcarnitines were infrequently detected in soleus (Fig.6).

Specific short-chain fatty acylcarnitines derived from amino acid metabolism (e.g., propionylcarnitine, malonylcarnitine, butyrylcarnitine, and valerylcarnitine) were unchanged with RW activity (Fig.6). The modest activity stimulus may not have been large enough to activate anaplerosis and alter protein metabolism. In further support of this hypothesis, only one of the 20 proteinogenic amino acids (i.e., glutamate in soleus) showed a change with RW activity (Fig.6). Note that an interaction with 16 h fasting is implicit to both groups, so RW activity was insufficient to enhance or dampen protein metabolism beyond the response to fasting. It would be interesting to test the effects of RW activity in older, sedentary, fasted sarcopenic rats with demonstrated alterations to the amino acid metabolomic footprint (Garvey et al. 2014).

Several glycerophospholipids (GPLs) increased in RW+ plantaris (Fig.6). GPLs are saturated long-chain glycerophospho-ethanolamines and -cholines created by the phospholipase-mediated hydrolysis of polyunsaturated phospholipids. GPLs are critical structural components of the plasma membrane that promote positive, or fluidizing, curvature to membranes (Fuller and Rand 2001). These data suggest that plantaris may adapt to RW activity by shifting its plasma and organellar membrane lipomic profiles over time, or in the fasted state, by acutely catabolizing GPLs for energy. Since various forms of physical activity increase intracellular autophagy, as also suggested by the increased LC3B-II/-I ratio in our study (Fig.5), it is possible that autophagy associated with RW activity contributed to the change in the GPL profile. Additional studies are needed to determine the source and function of GPLs in skeletal muscle.

Very few metabolomic changes were observed in glucose metabolism in our study (Table S1), although a separate targeted assay showed elevated fasting intramuscular glycogen with RW activity (Fig.5). It is interesting that a relative decrease of glycogenolytic intermediates (i.e., maltotriose and maltopentaose) was observed in RW+ rats (Fig.6). Our finding that intramuscular glycogen, a marker of anaerobic potential, was increased in the RW+ group might be viewed as surprising, since resting glycogen has been shown to increase glycolytic rate, at least in response to activity or contraction (Richter and Galbo 1985), and there were no differences across glycolytic intermediates in this study, at least at rest (Table S1). Alternatively, it might well be expected that a training effect from RW activity augmented glycogen loading. We also speculate that the accumulation of glycogenolytic intermediates in the RW− rats followed from impaired flux through the full glycolytic pathway, leading to an increase in glycogenolysis. Glycolysis may be more responsive in young rats due to the far increased amount of RW activity, in which increased GLUT4, hexokinase, and citrate synthase expression were observed in the plantaris muscle (Henriksen and Halseth 1995). We predict that the metabolomic profile of younger rats would show much greater perturbation of glycolytic metabolites.

Several important caveats regarding interpretation of data both from whole muscle groups and from fasted animals have been discussed prior (Garvey et al. 2014). Briefly, the SD rats used in this study were fasted for 16 h overnight prior to collection of tissue. Such fasting can cause changes in up to 50% of the metabolome of serum, urine, and liver, compared to ad libitum feeding (Robertson et al. 2011). Thus, molecular and metabolomic differences reported herein may not necessarily be observed postprandially or throughout ad libitum feeding. A second critical consideration is that skeletal muscles are metabolically heterogeneous tissues. The rat plantaris and gastrocnemius muscles predominantly comprised glycolytic MHC type IIB myofibers, whereas the soleus muscle predominantly contains oxidative MHC type I myofibers (Staron et al. 1999; Eng et al. 2008). The various outcomes in this study were measured in different muscles – grip strength in the forelimb, myofiber CSA in the lateral gastrocnemius, protein expression in the medial gastrocnemius, and metabolomic profiling of soleus and plantaris. Furthermore, the plantaris, although it has an MHC profile similar to the gastrocnemius, actually exhibits in vivo activation patterns more similar to the soleus (Hodson-Tole and Wakeling 2010).

In summary, we have shown that low-volume running wheel activity can induce significant biochemical and metabolomic changes in mixed hindlimb muscles of late middle-aged rats. Our results are consistent with periodic, chronic activation of the AMPK signaling pathway, leading to increases in markers of mitochondrial biogenesis, autophagy and negative regulation of muscle mass. These data promise to better define physiological boundaries for the emerging concept of healthy sedentarism beyond middle age.

## Supporting Information

**Table S1**. Metabolomics: Relative ratios of plantaris and soleus muscle biochemicals from 18- to 19-month-old SD rats with 8 weeks of voluntary running wheel access (RW+) or no access (RW−).
